# Electroencephalogram response in premature infants to different odors: a feasibility study

**DOI:** 10.1007/s12519-022-00609-2

**Published:** 2022-09-17

**Authors:** Janine Gellrich, Sandy Schlage, Veronika Messer, Valentin A. Schriever

**Affiliations:** 1 Abteilung Neuropädiatrie Medizinische Fakultät Carl Gustav Carus, Technische Universität, Dresden, Germany; 2 Klinik und Poliklinik für Kinder- und Jugendmedizin, Medizinische Fakultät Carl Gustav Carus, Technische Universität Dresden, Fetscherstrasse 74, 01307 Dresden, Germany; 3 Center for Chronically Sick Children (Sozialpädiatrisches Zentrum, SPZ), Charité - Universitätsmedizin Berlin, Berlin, Germany; 4 Department of Pediatric Neurology, Charité - Universitätsmedizin Berlin, Berlin, Germany

The importance of olfaction for newborns, especially for mother-associated odors is well known. Olfactory receptor neurons were found in preterm infants as early as 24–27 weeks of gestation [1]. Therefore, it can be assumed that the olfactory sense is functioning in preterm infants [2], but only very little is known about olfactory processing at this age. Most studies dealing with the processing of olfactory stimuli have been conducted on mature infants. However, in 2001 a research group demonstrated, using near-infrared spectroscopy, that a change in blood oxygenation of premature infants could be detected in the orbitofrontal cortex after olfactory stimulation with disinfectant and detergent [3]. Electroencephalogram (EEG) techniques have broadly been used to measure central odor processing in adults and children [4–6]. EEG has a high temporal resolution and is more stable to movement artifacts compared to magnetic resonance imaging (MRI). Brain activity measured by means of the EEG in premature infants is known to differ from that of term-born infants in terms of predominant frequency and amplitude. From the age of 28–29 weeks of gestation, a change in EEG to external stimuli can be observed [7]. The aim of the present study was to investigate the possibility of detecting central odor processing in premature infants by analyzing olfactory-induced EEG changes using Fast Fourier transformation (FFT) as an already established method in older term-born infants [6]. The previous study from our group demonstrated that EEG method and FFT analysis were feasible for mature infants and showed that EEG amplitudes in the δ frequency band differed significantly after the presentation of breast milk in comparison to an odorless control stimulus and farnesol odor, especially in frontal recording positions [6]. Physiologically, predominant EEG activity is in lower frequencies in preterm infant compared to term born or mature infants, leading to potentially higher susceptibility of the EEG recording to interference with the stimulus processing frequencies. Therefore, the aim of this study was to assess the transferability of the methods tested in mature infant to preterm infants.

More specifically, the aim of this feasibility study was to investigate central odor processing in premature infants using EEG analyses with a focus on whether (1) a study set up developed for older infants is transferable to preterm infants [6] and (2) different olfactory stimuli result in different EEG changes of premature infants.

In this analysis, six premature infants (two boys, four girls) between the postnatal age of 16 and 34 (27.00 ± 6.03 days) days were included. The infants were recruited from the intermediate care ward of the pediatric department. All infants were completely oral fed. All infants were born prematurely (33 ± 1.98, range 31–35 gestational weeks). Their birthweight was on average 1630 g, and birth size was 42.7 cm. No EEG changing medication was applied to any of the infants. All participants were able to finish the EEG.

EEG setup, data acquisition and processing were performed using a small transportable computer-controlled olfactometer [8]. Stimuli were administered using a nasal cannula. The parameters were collected identically in a previous study, which investigated similar parameters in older term newborns [6]. Three odors have been used: breast milk of their own mother, farnesol diluted 1:10 with 1,2-propranediol and vanilla (4-hydroxy-3-methoxybenzaldehyd) with a concentration of 3 g per 100 mL of 1,2-propanediol. Farnesol, 1,2-propranediol and vanilla were purchased from Sigma-Aldrich, Deisenhofen, Germany. Before the study was conducted, care was taken to ensure that the odors presented were all isointense. The airflow of the olfactometer was fixed at 1 L/minute for each nostril. Odors were administered under constant airflow. Stimulus duration was 1 s followed by an ISI (interstimulus interval) of 20 s. Odor presentation was pseudo-randomized in blocks of three to five stimuli of the same odor. Based on previous work, 90 stimuli which included 30 stimuli of each odor, were presented [6]. Odorless air was presented between odor presentations to avoid adaptation.

EEG electrodes were used according to the international 10–20 system [9]. The following recording sites were used: F3, Fz, F4, C3, Cz, C4, P3, Pz, P4. For EEG recording a Natus Delta Med system (Natus Medical Incorporated) has been used according to the following settings: sampling frequency 256 Hz, reference Fpz, gold cup electrodes (Genuine Grass, Natus Medical Incorporated, Pleasanton, CA 94566 USA). To minimize the artifacts resulting from eye or muscle movements and blinking, attempts were made to measure the EEG during the infant’s morning nap or while the infant was in a calm resting state.

Letswave 6 toolbox for MATLAB (http://nocions.github.io/letswave6; The MathWorks, Natik, Massachusetts 01760 USA) has been used for data analysis. The EEG recordings were pre-processed using a high-pass frequency filter of 0.5 Hz. Segmentation of continuous EEG recordings to epochs of 4000 ms has been done, beginning from the 1000 ms before stimulus onset. Following a low-pass frequency filter of 20 Hz, epochs were sorted according to the olfactory stimulus. For baseline correction, a reference interval of 1000 ms before stimulus onset was used. Because of different EEG amplitudes between individual infants, the individual amplitude criterion was used for artifact rejection. Epochs above double standard deviations from the mean of amplitude were excluded. In addition, visual artifact rejection was applied to exclude blinking artifacts. Based on a previous study, there must be a minimum of eight artifact-free epochs for analyzing central olfactory processing by means of EEG [10]. About 73% of the farnesol, 62% of the milk and 68% of the vanilla epochs could be included in the analysis after artifact rejection. FFT was applied on a post-stimulus interval of 400–2000 ms taking amplitude as the output variable for averaged epochs. Three frequency bands were analyzed using FFT separately: delta (*δ*, 0.5–4 Hz), theta (*θ*, 4–8 Hz) and alpha (*α*, 8–13 Hz). In addition, EEG epochs 7000–3000 ms pre-stimulus onset, during the ISI were used as a control condition. The EEG processing steps also were applied to the 90 epochs of the control condition and then were summed up. Amplitudes were excerpted from the FFT analysis for each of the frequency bands and odor conditions for every infant. Acquisition of EEG data was possible in the examined preterm infants, and odor presentation showed a significant change in amplitude in FFT analysis compared to the odorless control condition using Kruskal-Wallis testing (*P* < 0.001). According to Cohen, it is a low effect with *r* = 0.11 [11, 12]. The main effect of the EEG frequency band on the EEG amplitude could be observed (*P* < 0.001) showing higher amplitudes in lower frequencies (*δ*: 1.43 ± 1.25 µV; *θ*: 0.81 ± 1.03 µV; *α*: 0.36 ± 0.42 µV) [mean ± standard error (SE)] as it is reported physiologically. Furthermore, the interaction between olfactory stimulus and frequency on amplitude could be displayed (*P* = 0.008). Bonferroni-adjusted post-hoc analysis revealed significant differences in *δ* [*P* < 0.001; 95% confidence interval (CI) 0.591−1.161] and θ frequency bands (*P* < 0.001; 95% CI 0.343−0.914) between odor stimulus and control while for the band only a trend (*P* = 0.093) could be displayed. There is no interaction in regard to the position of electrodes (frontal, central, parietal) and olfactory stimulus.

The main effect of the olfactory stimulus on the amplitude of EEG showed a significant difference between the different odors using Kruskal-Wallis testing (*z* = 76.969; *P* < 0.001), which again corresponds to a rather low effect (*r* = 0.12) according to Cohen's test [11, 12]. Furthermore, pairwise testing using post-hoc Dunn-Bonferroni analysis displayed a significant difference between each presented odor and the control condition (control 0.43 ± 0.451 µV, breast milk 0.81 ± 0.81 µV, vanilla 0.85 ± 0.9 µV, farnesol 1.38 ± 1.54 µV, all *P* < 0.001). While no significant difference in EEG amplitude could be observed comparing breast milk and vanilla odor stimulation (*P* = 0.778), a significant difference in regard to EEG amplitudes could be seen after breast milk compared to farnesol odor stimulation (*P* = 0.039). This significance was not evident comparing EEG amplitudes after vanilla and farnesol stimulation (*P* = 0.072). Here only a trend could be detected.

An interaction between odor stimulus and EEG frequency band on the EEG amplitude was observed (*P* = 0.006). Post-hoc analysis revealed an effect of odor stimulus on the EEG amplitude within the δ (*P* < 0.001) and the θ frequency bands (*P* < 0.001), but not in the α frequency band (*P* = 0.125). In line with the findings presented above for the three frequency bands together, significant differences were found within δ and θ frequency band between the EEG amplitude after each of the three odor stimuli in comparison to the control condition (all *P* < 0.001) (Fig. 1). Significant difference in EEG amplitudes between the three different odors that were presented could not be found for the three frequency bands separately.

**Fig. 1:**
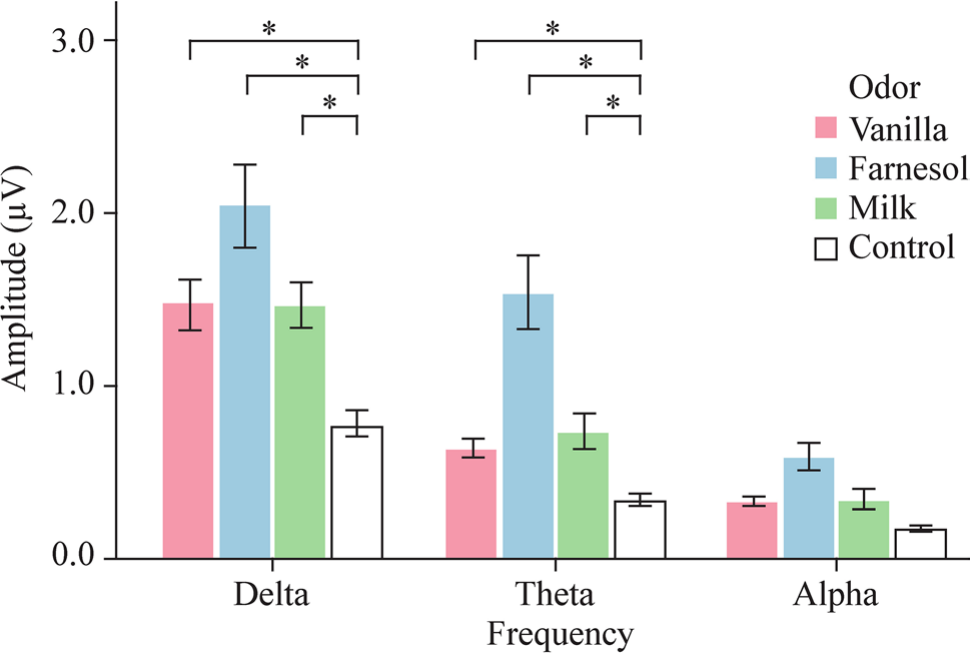
The graph is showing the effect of the stimulus on the electroencephalogram amplitude within the different frequency bands for each odor displayed by mean and double standard error. Significant difference was found between EEG amplitudes after milk, farnesol and vanilla odor in comparison to the control condition within *δ* and *s* frequency (*). Alpha level was set to 0.05

The aim of this feasibility study was to investigate central odor processing in premature infants using EEG analyses. The results showed that a transfer of the study design to preterm infants is possible and that an FFT analysis is suitable for EEG data analyses in this age group after olfactory stimulation. The results showed a significant change in EEG amplitude after odor presentation in comparison to the odorless control condition. This was true for δ and the θ frequency bands. The localization of the EEG electrodes did not have an influence on our study population of preterm infants. This result is a discrepancy relative to a previous study in older term-born infants [6]. The significant difference in change of EEG amplitude after different olfactory stimuli (farnesol *vs.* milk), with higher EEG amplitude after farnesol odor presentation, might indicate differences in central odor processing in premature infants as a neuronal correlate for odor discrimination. Farnesol, as a floral non-food associated and most likely unknown odor, is clearly different from breast milk and vanilla odor and therefore might lead to arousal and increase attention in premature infants [11–13]. EEG amplitudes after olfactory stimulation in our preterm infants were lower compared to EEG amplitudes of term-born older infants in a previous study [6]. This appears to be physiological for the age of 28 to 34 gestational weeks and results from tracé discontinuation and alternate [14].

In conclusion, assessment of central odor processing in premature infants at 31–35 weeks of gestation by means of EEG is feasible with the used study design and settings. More detailed studies with larger cohorts are needed and warranted to better understand the processing of olfactory information in the brain of preterm infants.

## Data Availability

Data regarding patients’ information can not be published according to German right to data protection. Data can be available once the request comply with the policy.

## References

[1] Chuah MI, Zheng DR (1987). Olfactory marker protein is present in olfactory receptor cells of human fetuses. Neuroscience.

[2] Schaal B, Hummel T, Soussignan R (2004). Olfaction in the fetal and premature infant: functional status and clinical implications. Clin Perinatol.

[3] Bartocci M, Winberg J, Papendieck G, Mustica T, Serra G, Lagercrantz H (2001). Cerebral hemodynamic response to unpleasant odors in the preterm newborn measured by near-infrared spectroscopy. Pediatr Res.

[4] Huart C, Eloy P, Collet S, Rombaux P (2012). Chemosensory function assessed with psychophysical testing and event-related potentials in patients with atrophic rhinitis. Eur Arch Otorhinolaryngol.

[5] Schriever VA, Boerner C, Mori E, Smitka M, Hummel T (2015). Changes of olfactory processing in childhood and adolescence. Neuroscience.

[6] Gellrich J, Breuer AS, Han P, Güdücü C, Hummel T, Schriever VA (2021). Central nervous system processing of floral odor and mother’s milk odor in infants. Chem Senses.

[7] Vecchierini MF, André M, d’Allest AM (2007). Normal EEG of premature infants born between 24 and 30 weeks gestational age: Terminology, definitions and maturation aspects. Neurophysiol Clin Neurophysiol.

[8] Schriever VA, Körner J, Beyer R, Viana S, Seo HS (2011). A computer-controlled olfactometer for a self-administered odor identification test. Eur Arch Otorhinolaryngol.

[9] Klem GH, Lüders HO, Jasper HH, Elger C (1999). The ten-twenty electrode system of the international federation. The international federation of clinical neurophysiology. Electroencephalogr Clin Neurophysiol Suppl.

[10] Hummel T, Klimek L, Welge-Lüssen A, Wolfensberger G, Gudziol H, Renner B (2000). Chemosensorisch evozierte Potentiale zur klinischen Diagnostik von Riechstörungen. HNO.

[11] Cohen J (1992). A power primer. Psychol Bull.

[12] Aoyama S, Toshima T, Saito Y, Konishi N, Motoshige K, Ishikawa N (2010). Maternal breast milk odour induces frontal lobe activation in neonates: A NIRS study. Early Hum Dev.

[13] Bartocci M, Winberg J, Ruggiero C, Bergqvist LL, Serra G, Lagercrantz H (2000). Activation of olfactory cortex in newborn infants after odor stimulation: a functional near-infrared spectroscopy study. Pediatr Res.

[14] André M, Lamblin MD, d’Allest AM, Curzi-Dascalova L, Moussalli-Salefranque F, Nguyen STT (2010). Electroencephalography in premature and full-term infants developmental features and glossary. Neurophysiol Clin Neurophysiol..

